# The Cotton Mealybug Is Spreading along the Mediterranean: First Pest Detection in Italian Tomatoes

**DOI:** 10.3390/insects12080675

**Published:** 2021-07-27

**Authors:** Michele Ricupero, Antonio Biondi, Agatino Russo, Lucia Zappalà, Gaetana Mazzeo

**Affiliations:** Department of Agriculture, Food and Environment, University of Catania, 95123 Catania, Italy; antonio.biondi@unict.it (A.B.); agarusso@unict.it (A.R.); lzappala@unict.it (L.Z.); gamazzeo@unict.it (G.M.)

**Keywords:** alien species, biological invasions, Coccinea, cotton mealybug, Homoptera, pseudococcidae, scale insects, sustainability

## Abstract

**Simple Summary:**

Nowadays, globalization causes a series of negative consequence for the sustainability of agricultural systems, such as solanaceous cultivations. Here, we report for the first time the presence of an invasive polyphagous mealybug, *Phenacoccus solenopsis* Tinsley (Hemiptera: Pseudococcidae), in Italian solanaceous crops, i.e., tomato and bell pepper. For this, we analyzed at the morphological and molecular level various specimens collected in Sicily during fall 2020. A phylogenetic analysis conducted comparing an ≈800 bp portion of the mitochondrial genome of several worldwide populations, suggests that the introduced population might originate from Asia. This study represents the first step toward establishing a *P. solenopsis* monitoring and sustainable control program in Europe.

**Abstract:**

The cotton mealybug *Phenacoccus solenopsis* Tinsley (Hemiptera: Pseudococcidae) is an extremely polyphagous invasive pest that can cause serious damages to cultivated plants. The pest is native to America but invaded Asian and Mediterranean countries during the last decades. Tomato (*Lycopersicon esculentum* Mill., Solanaceae) is an economic relevant crop worldwide and its production can be threatened by numerous insect pests including *P. solenopsis*. We recorded for the first time *P. solenopsis* in association with tomato in greenhouse crops and urban landscapes in Sicily (Italy) during the fall season in 2020. The species was identified as *P. solenopsis* based on the morphological characters and DNA amplification of an ≈800 bp portion of mitochondrial cytochrome oxidase subunit I (mtCOI) gene. The phylogenetic analysis among the obtained *P. solenopsis* mtCOI sequences with those already available in GenBank suggests Asian countries as a potential source of new introduction. This is the first record of *P. solenopsis* attacking tomato plants in Italy and may represent a potential threat for tomato production in Europe and nearby countries. For this reason, actions should be taken to avoid the uncontrolled spread of this alien species.

## 1. Introduction

Biological invasions are one of the major challenges facing agriculture in the era of the global economy [1,2]. Invasive arthropod pests of economic relevance cause yield losses, increase costs for their control and often lead to pesticide overuse which disrupts existing ecosystem services [3,4,5]. Mealybugs (Hemiptera: Pseudococcidae) are typical invasive pests in many areas of the globe because of their small size and cryptic behavior [6], and they can cause serious economic damages in many cultivated crops [7].

*Phenacoccus solenopsis* Tinsley (Hemiptera: Pseudococcidae) is a highly polyphagous sap-feeding herbivore that has been reported on over 200 host plant species belonging to approximately 60 botanical families [8,9]. This mealybug feeds on phloem sap mainly on the aboveground plant parts and releases honeydew and waxy secretion as excretory products. The species has a mutualistic relationship with ants (Hymenoptera: Formicidae) and its diffusion is mediated by wind, although its widespread distribution has been mainly related to human activities [10,11]. The identification of *P. solenopsis* is generally carried out through morphological observation; however, this method is routinely supported by DNA characterization [12]. The mealybug can be identified through the presence of dark spots on the dorsum of the female body; however, some specimens can be unpigmented being thus misidentified with *P. solani* [13]. Other morphological and molecular variations among geographical populations of *P. solenopsis* have been also recognized [13,14,15,16], and these differences have been attributed to environmental conditions and/or host plants [17].

*Phenacoccus solenopsis*, whose description dates back in New Mexico (USA) in the late 19th century [18], has been reported outside its native range in more than 50 locations worldwide over the last decades. This invasive pest has established in the Afrotropical, Australasian, Nearctic, Neotropical, and Indomalayan realms, and it is considered one of the most devastating pests of cotton in Asia, notably in China, India, and Pakistan [19]. Within the Palaearctic region, the species has been reported during the last ten years in the Mediterranean basin including Algeria, Canarian islands, Cyprus, Egypt, Israel, Turkey and Saudi Arabia [9,20,21,22,23,24,25,26,27]. In a recently published opinion by EFSA Panel on Plant Health, the species is reported as being present in Crete on tomato plants according to a personal communication [28]. Although in these regions, *P. solenopsis* has been primarily recorded on wild hosts and ornamentals (e.g., *Ibiscus* sp. and *Lantana* sp.), in Israel and Egypt the mealybug has become a serious pest in few cotton fields and solanaceous protected crops, namely in bell pepper and tomato greenhouses [24,25].

Tomato (*Lycopersicon esculentum* Mill., Solanaceae) is the most economically relevant horticultural crop worldwide with 4.7 million cultivated hectares and a global production exceeding 182 million tons [29]. This crop is threatened by a large number of invasive pests such as The South American tomato pinworm, *Tuta absoluta* (Meyrick) (Lepidoptera: Gelechiidae), that severely affected tomato production in the Palaearctic region in the last decade [30,31]. Mediterranean countries play a central role in the supply chain of European tomato; thus, the early detection of new emerging pests that pose risk for the European tomato production is of paramount importance.

In this article, we provide the first evidence of *P. solenopsis* in different protected crops and urban landscapes in Sicily, the southernmost main island of Italy, located in the central Mediterranean basin, that serves as a hotspot for the European tomato industry [32]. The mealybug specific identification was carried out by using morphological features together with DNA characterization of the mitochondrial cytochrome oxidase subunit (mtCOI). We also investigated the genetic relationships of *P. solenopsis* by using the mtCOI gene from the specimens that we collected and other similar accessions originating from different world regions to identify the source of invasion of this invasive mealybug into Italy. Our findings alert the presence of *P. solenopsis*, highlighting the importance of early detection and the need for precautionary control measures against this invasive mealybug pest.

## 2. Materials and Methods

### 2.1. Insect Sampling

We carried out occasional samplings of mealybugs between October and November 2020 in protected crops and urban parks in Sicily, namely in four sites located on the southern border of the island. The aboveground part of infested plants (cultivated and wild) was inspected, female scales were collected with a soft paintbrush and kept in 90% ethanol inside plastic vials for identification in the laboratory. We also sampled ants directly associated with the mealybugs as described above for the species identification. Pictures of the infested plants were taken by using a Nikon D3100 Digital Camera (Nikon Corporation, Tokyo, Japan).

### 2.2. Morphological Identification

The morphological identification was conducted through the keys proposed by Williams and Granara de Willink [33], Granara and Szumik [34] and Hodgson et al. [17]. The collected adult females and nymphs were morphologically identified directly using a stereomicroscope or mounted on slides to be examined using a compound microscope.

From each sample, 5 adult females were slide-mounted in Canada balsam using the method described by Williams and Watson [35]. Pictures were taken with a stereomicroscope Leica Ez 4 D (Leica Microsystems, Heerbrugg, Switzerland) with an integrated digital camera. Slide-mounted specimens are deposited at the Scale Insect Collection of the Department of Agriculture, Food and Environment of the University of Catania (Italy).

### 2.3. Molecular Identification

We conducted the molecular identification of the collected scales by sequencing the amplified mtCOI gene fragment in the mealybug DNA as follows. Briefly, a total of 3 mealybug females per each sampled location (see Table 1) were crushed with a sterile pestle in a 2 mL Eppendorf tube and subjected to DNA extraction using the E.Z.N.A.^®^ Tissue DNA Kit (Omega Bio-tek, Inc., Norcross, GA, USA). In the DNA extraction, we included also samples of the citrus mealybug *Planococcus citri* (Risso) (Hemiptera: Pseudococcidae) as DNA positive control and a negative control without DNA. Universal primer pairs C1-J-2195 (alias Jerry, 5′-TTGATTTTTTGGTCATCCAGAAG-3′) and TL2-N-3014 (alias Pat, 5′-TCCAATGCACTAATCTGCCATATTA-3′) were used to amplify the expected ≈800 bp of the mtCOI targeted region [36]. PCR was carried out following the protocol suggested by Cavalieri et al. [37]. The reactions were performed in 20 μL volumes with 0.85X of FailSafeTM PCR 2X PreMix F (Lucigen Corporation, Middleton, WI, USA), 0.5 μM of each primer 10 μM, 1.5 U of Taq DNA Polymerase 5U (Invitrogen, Thermo Fisher Scientific, Waltham, MA, USA) and 2 μL of DNA template. The cycling conditions were as follows: 96 °C for 5 min, 35 cycles at 96 °C for 45 s, 45 °C for 1 min, 72 °C for 1 min, followed by a final cycle at 72 °C for 10 min. Reactions and cycling conditions were carried out in the thermal cycler Eppendorf Mastercycler^®^ EP Gradient S.

The amplification success of PCR products was checked by electrophoresis using 1% agarose gel. When DNA bands of the expected size (≈800 bp) were visualized in the gel, the remaining PCR products were shipped to a BMR Genomics (Padova, Italy) sequencing service that purified and sequenced the PCR products through Sanger’s method. Thus, the obtained coding regions were thus manually checked for errors, trimmed for low quality, and aligned to reference sequences from the National Center for Biotechnology Information (NCBI) GenBank^®^ through Basic Local Alignment Search Tool (BLAST) sequence analysis tool for the species identification [38]. A representative sequence from each sampling was deposited in GenBank (accession numbers are given in Table 1).

### 2.4. Phylogenetic Analyses

Evolutionary relationships among *P. solenopsis* isolates from native (American) and invasive (Mediterranean and Asian) origins were estimated by constructing phylogenetic trees based on mtCOI sequences derived from our samples (Table 1) and those retrieved in GenBank in January 2021 (Table S1). Sequence records were screened out for their coverage within the region amplified by C1-J-2195 and TL2-N-3014 primers and those containing scarce, ambiguous or repeated information about the sampling location were discarded. Thus, thirty selected nucleotide sequences were aligned with the MUSCLE algorithm [39], and their ends were trimmed to produce 731bp alignments in Unipro UGENE version 1.26.1 [40]. We also screened translated mtCOI sequences for stop codons to exclude any possible mitochondrial pseudogenes that often occur in invertebrates. *Maconellicoccus hirsutus* (Green) (Hemiptera: Pseudococcidae) isolate mtCOI sequence (GenBank accession number EU267199.1) was included in the dataset as an outgroup. The evolutionary history was inferred by using the Maximum Likelihood (ML) method and Kimura 2-parameter model [41]. The initial tree for the heuristic search was obtained automatically by applying Neighbor-Join and BioNJ algorithms to a matrix of pairwise distances estimated using the Maximum Composite Likelihood (MCL) approach and then selecting the topology with superior log likelihood values. This analysis involved 31 nucleotide sequences. Codon positions included were 1st + 2nd + 3rd + Noncoding. The reliability of the branches was estimated using 1000 bootstraps. Evolutionary analyses were conducted in MEGA X [42,43].

## 3. Results

### 3.1. Insect Sampling

**Figure 1:**
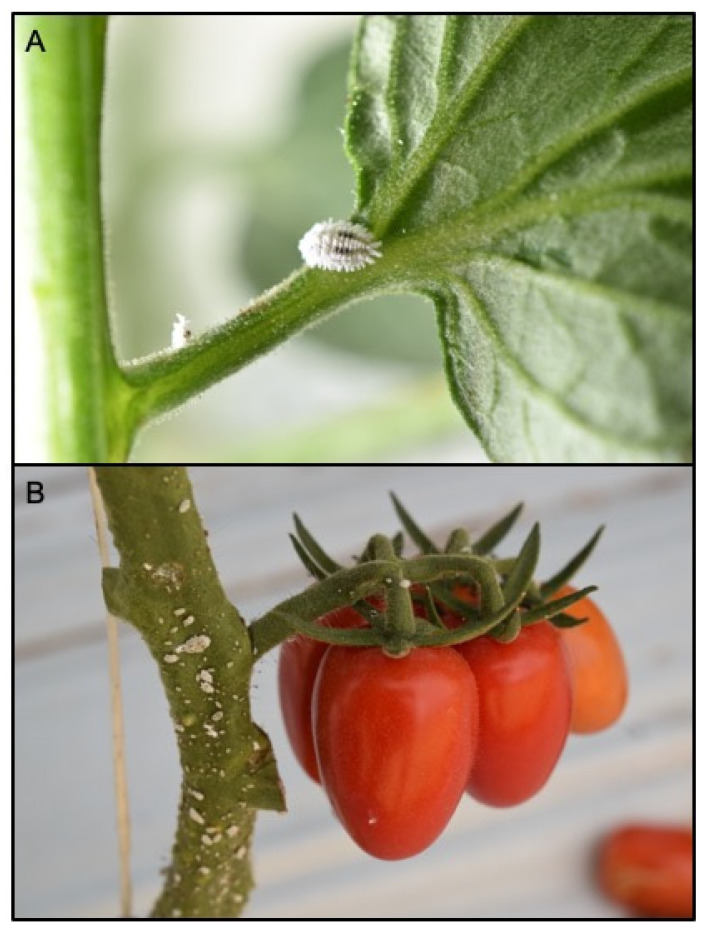
*Phenacoccus solenopsis* adult female (**A**) and mixed instars (**B**) on a twig of tomato plant in greenhouse tomato crop.

Among the different surveyed sites in Sicily between October and November 2020, *P. solenopsis* was found in three localities (Table 1). Mealybug individuals were found on the stems, branches and root collar of conventional cherry-type tomato greenhouses (Figure 1) and in tunneled bell pepper in Marina di Ragusa and Palma di Montechiaro, respectively. Lantana bushes and hibiscus plants in two different urban parks of Catania were similarly found infested by the mealybug pest. Collected samples were identified as *P. solenopsis* through morphological and molecular analysis.

### 3.2. Morphological Identification

**Figure 2:**
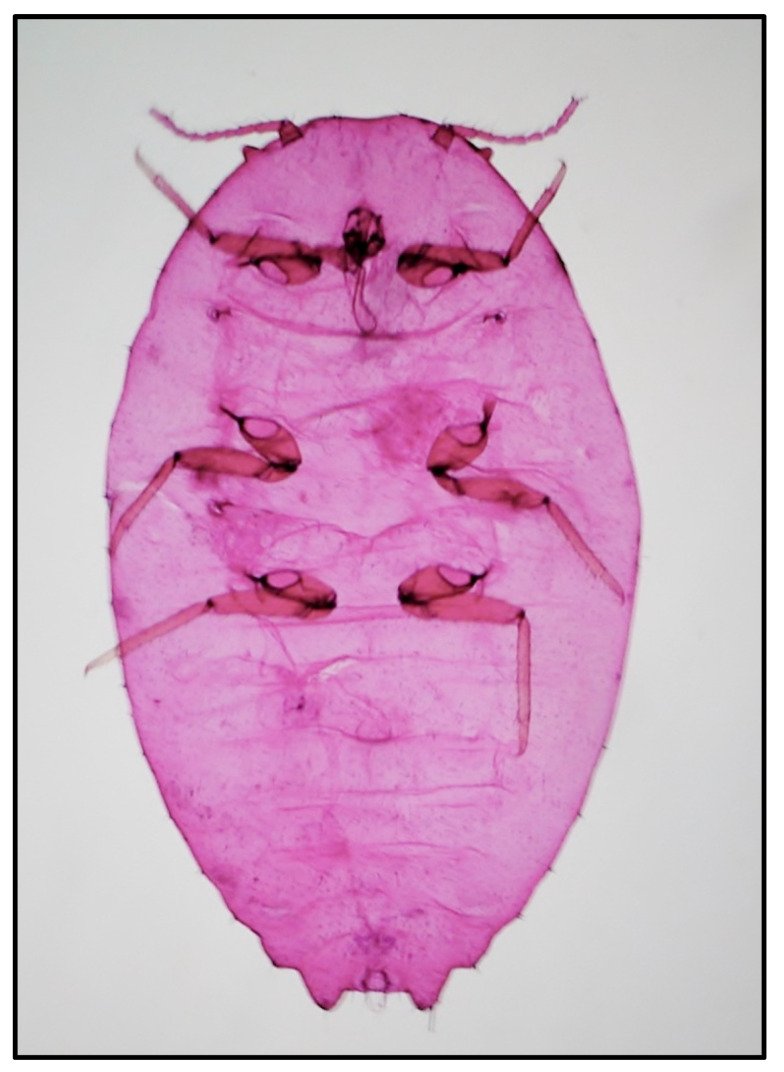
Slide-mounted female of *Phenacoccus solenopsis*.

**Figure 3:**
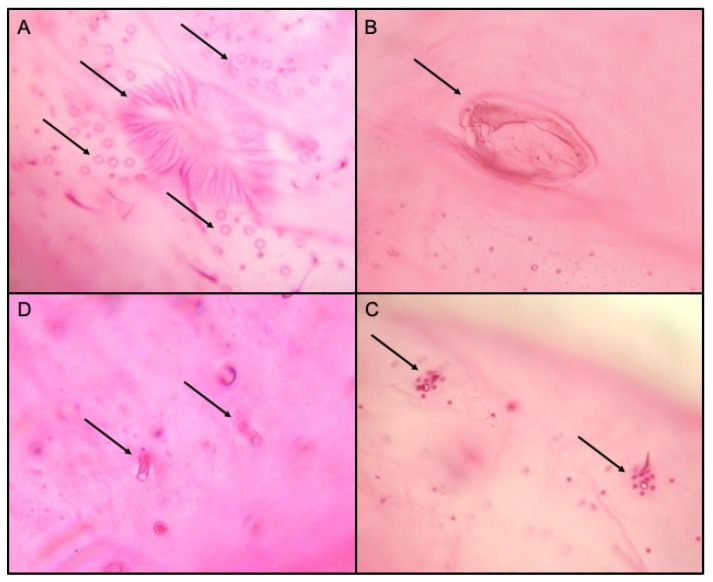
Morphological features of *Phenacoccus solenopsis*, the vulva and multilocular disc pores (**A**), circulus (**B**), cerari (**C**) and oral collar tubular ducts (**D**).

The external morphological examination revealed that adult females had two dark stripes on either side of a middle body ridge, short waxy filaments around the body, and quarter-length anal filaments (Figure 1). The slide-mounted female shows an oval body about 3.35 ± 0.14 mm long and 2.2 ± 0.14 mm wide; antennae are usually nine segmented, *circulus* present, oval, occasionally slightly constricted laterally, and variable in size; *cerarii* numbering 18 pairs each with two conical setae and trilocular pores without auxiliary setae; oral collar tubular ducts on venter only; quinquelocular pores absent; multilocular disc pores absent dorsally, present medially on the venter of segments VI–IX (rarely also one or two on V), also usually present submarginally on some abdominal segments (Figure 2 and Figure 3). The specimens collected in Sicily showed a range per side in multilocular disc pores of 7–8, 11–11, and 8–12 in the samples coming from Palma di Montechiaro, Marina di Ragusa and Catania, respectively.

Since *P. solenopsis* is very similar to *P. solani*, differences among the two species were highlighted. According to Williams [44], the species we identified as *P. solenopsis* had a more flaccid circulus and the multilocular disc pores present on the anterior edges of the posterior abdominal segments, whilst in *P. solani*, the circulus is more rounded, and the multilocular disc pores are restricted to the posterior margins of the abdominal segments anterior to the vulva.

Ant species associated with *P. solenopsis* were identified as *Tapinoma magnum* (Mayr) (Hymenoptera: Formicidae).

### 3.3. Molecular Identification

Based on mtCOI sequence data, the individuals sampled in geographically separated areas in Sicily and from different hosts shared the same haplotype. Subsequently, we confirmed the morphological identification of *P. solenopsis* through mtCOI fragment amplification from genomic DNA, followed by direct sequencing and BLAST searches. The resulting sequences were aligned to reference sequences from NCBI compared with publicly available data on GenBank and yielded an identity score of 100% and E-value = 0.0 with *P. solenopsis* isolates from China (Accession number KF878039.1), India (Accession number KC985430.1) and Pakistan (Accession n. KF442955.1).

### 3.4. Phylogenetic Analysis

We analyzed a total of 30 mtCOI sequences, four of which were sequenced in this study.

**Figure 4:**
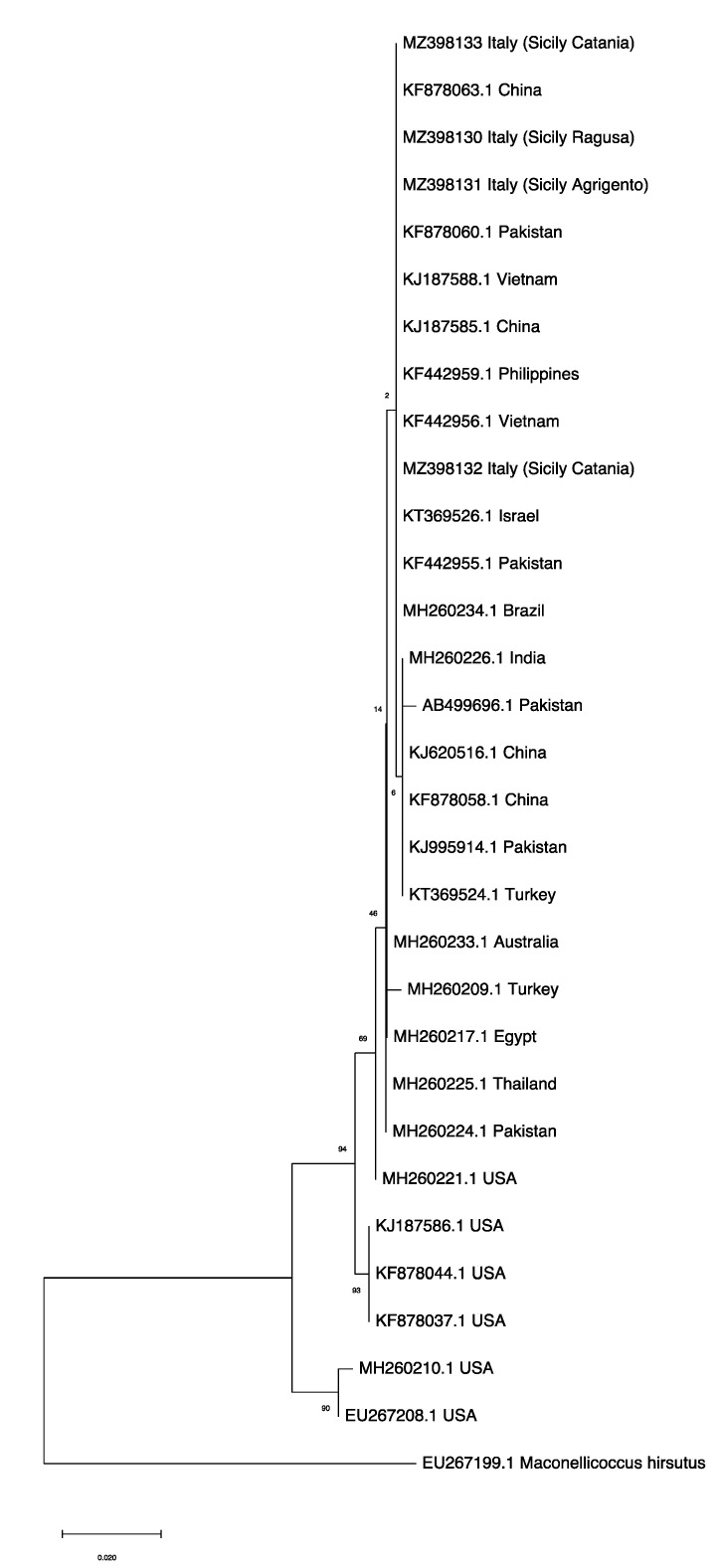
Maximum Likelihood tree, with bootstrap values, based on mtCOI sequences of *Phenacoccus solenopsis* collected in Sicily clustering with publicly available accessions from invaded (Australia, Brazil, China, Egypt, India, Pakistan, Philippines, Thailand, Turkey, Vietnam) and native (USA) countries retrieved in GenBank (Table S1). *Maconellicoccus hirsutus* (Accession n. EU267199.1) was used as an outgroup. The tree with the highest log likelihood (−1550.08) is shown. The percentage of trees in which the associated taxa clustered together is shown next to the branches. The tree is drawn to scale, with branch lengths measured in the number of substitutions per site.

Among them, *P. solenopsis* accessions came from different world countries including its native area (USA) and invaded areas (Table S1). According to the phylogenetic analysis, the ML tree included two main distinct clades: one clade consisted of samples from the USA and another clade grouped accessions from different *P. solenopsis* invaded areas (Figure 4). Notably, both clades shared a common ancestor derived from the USA. Nevertheless, the samples of *P. solenopsis* we sequenced (MZ398130; MZ398131; MZ398132; MZ398133) clustered together with isolates coming from different world regions (Brazil, China, Israel, Pakistan, Philippines, and Vietnam).

## 4. Discussion

*Phenacoccus solenopsis* is a sap-feeding herbivore native to Americas characterized by an extreme polyphagia that spread eastwards invading more than 50 countries during the last 30 years, becoming the major insect pest of cotton in Asia [19]. We recorded for the first time *P. solenopis* in Sicily on different host urban plants and protected crops including solanaceous plants such as tomato and bell pepper.

Morphological features were used to primarily assess the identity of the invasive species by using together several keys [33,34,35]. *Phenacoccus solenopsis* shows considerable morphological variations in the number of multilocular disc pores and oral collar tubular ducts on abdominal segments induced by environmental conditions and hosts [14,17]; according to our observations, the specimens found in Sicily have shown similarity to the group of *P. solenopsis* described by Hodgson et al. [17] for India, Pakistan and Taiwan. Differences between *P. solenopsis* and *P. solani* were also highlighted. The latter was already recorded in Sicily in 1999 [45], and these two congeneric species are very difficult to discriminate morphologically due to the variability of some of the traits used in morphological identification keys (e.g., the shape of the circulus, the number of antennal segments, and the distribution of multilocular disc pores) [34]. However, according to the key proposed by Williams [44], the samples we collected had a more flaccid circulus, and the multilocular disc pores were present on the anterior edges of the posterior abdominal segments, being thus recognized as *P. solenopsis*. Moreover, since Zhao et al. [13] have shown pigmented and non-pigmented *P. solenopsis* between samples from China, our observations clearly suggested that all our surveyed samples matched the pigmented variation. Lastly, in contrast to Thomas and Ramamurthy [14] who shown morphological variations in *P. solenopsis* specimens from different host plants, we did not observe any differences among the collected samples from different hosts.

We corroborated the mealybug identity through DNA amplification of the mtCOI fragment that is routinely used for insect discrimination at the species level. Other authors took into consideration such an approach for validation of this species with positive results [12,13,14,15,16]. We did not highlight molecular differences among *P. solenopsis* specimens surveyed in different host plants in accordance with a previous study conducted in Asia [14]. One goal of this study was to estimate the possible source population that has recently invaded Italy. According to our phylogenetic analyses, data suggested that the source of introduction of the sampled specimens might have originated from Asian populations rather than from the USA as also supported by morphological observations. However, the source of introduction of *P. solenopsis* into Italy might also derive from Mediterranean countries through which an intense trade is present, but no differences were highlighted by the analyses. Our findings are similar with previous studies wherein *P. solenopsis* origin was investigated using the mtCOI gene. Ahmed et al. [15] proved that the source of invasion for China, Pakistan, India and Vietnam likely derived from two by nine Asian haplotypes rather than those belonging to the American group. Wu et al. [16] asserted that the invasion of *P. solenopsis* into China may have originated from Pakistan rather than the USA. Nonetheless, because molecular data provide evidence that *P. solenopsis* may comprise a cryptic species complex, more samples from different world regions and more types of markers to analyze the genetic differentiation of the species should be considered to understand the invasion history and invasion pattern of *P. solenopsis* worldwide [46,47].

We provide the first evidence of *P. solenopsis* in different protected crops and urban landscapes in Sicily, and this record raises serious concerns because of the high risk of spread of this pest in the Northern Mediterranean basin. In this regard, Italy represents a suitable region for the biological success of invasive mealybugs, namely for its climatic conditions and geographical position, as demonstrated by the establishment of many *Phenacoccus* species that originated from South America [48]. Thus, surveillance of *P. solenopsis* is primarily required to identify its host plants and its current distribution in the detection site and the nearby areas. This aspect becomes particularly important because *P. solenopsis* can resist starvation, thus increasing its chances to survive on non-plant materials and be successfully transported into new areas [47]. Similarly, life history studies on host plants under different environmental circumstances could help to predict the diffusion of *P. solenopsis* in the newly invaded territory [11]. The species may further enter the EU territory with imported fresh fruit, vegetables, flowers, and plants for planting. Conversely, some plants, which are also *P. solenopsis* host plants, are prohibited from entering the EU as plants for planting by the EU regulation in force (Commission Implementing Regulation (EU) 2019/2072); however, no specific requirements are established in relation to *P. solenopsis*.

So far, more than 50 natural enemies, including both predators and parasitoids, have been already recorded attacking *P. solenopsis* on its wide distribution range, with coccinellid predators (Coleoptera: Coccinellidae) being the most relevant group [47]. Therefore, the recruitment of indigenous natural enemies that colonize *P. solenopsis* in the new invaded area should be carried out with the aim of finding potential biocontrol agents of this invasive pest. Together with biological control agents, environmentally friendly control methods should be investigated, with naturally derived pesticides as core tools [49]. Finally, studies on ants associated with the mealybug are also necessary because experimental evidence highlighted that the mutualism between *P. solenopsis* and *Solenopsis invicta* (Hymenoptera: Formicidae) may foster the invasion success of both species [10].

## Figures and Tables

**Table 1:** Location, sites, and host plants where *Phenacoccus solenopsis* was found in Sicily between October and November 2020.

Location	Sites		Host Plant	Date	NCBI Accession Number
	Latitude (N)	Longitude (E)			
Marina di Ragusa	36°47′11.7″	14°34′21.1″	*Lycopersicon esculentum* Mill. (Solanaceae) *Portulaca oleracea* L. (Portulacaceae) *Parietaria* sp. L. (Urticaceae) *Sesamum indicum* L. (Pedaliaceae)	22 October 2020	MZ398130
Palma di Montechiaro	37°11′37″	13°45′46″	*Capsicum annuum* L. (Solanaceae)	5 October 2020	MZ398131
Catania	37°31′18.0″	15°05′49.8″	*Hibiscus* sp. L. (Malvaceae)	4 Novenber 2020	MZ398132
	37°32′09.9″	15°04′06.1″	*Lantana camara* L. (Verbenaceae)	25 Novenber 2020	MZ398133

## Data Availability

The mitochondrial cytochrome oxidase I (COI) sequences generated in this study were then deposited in GenBank, accession numbers MZ398130-133.

## Supplementary Materials

The following are available online at https://www.mdpi.com/article/10.3390/insects12080675/s1, Table S1: Selected Phenacoccus solenopsis mtCOI sequences retrieved in GenBank (January 2021) used for phylogenetic analyses.
