# Supplementary material for: The Cotton Mealybug Is Spreading along the Mediterranean: First Pest Detection in Italian Tomatoes
Source: Insects. 2021 Jul 27;12(8):675. doi: 10.3390/insects12080675 (PMC8396508; doi:10.3390/insects12080675)
Supplement: Supplementary file 1 [file insects-12-00675-s001.zip › insects-1289484-supplementary.pdf]

Table S1. Selected *Phenacoccus solenopsis* mtCOI sequences retrieved in GenBank (January 2021) used for phylogenetic analyses.

Location	Host plant	NCBI Accession number
China: Guangdong	*Justicia gendarussa* (Acanthaceae)	KF878063.1
Pakistan	Unknow	KF878060.1
China: Guangxi	*Justicia gendarussa* (Acanthaceae)	KF878058.1
USA: California	Unknow	KF878044.1
USA: California	Unknow	KF878037.1
Brazil	Unknow	MH260234.1
Australia	Unknow	MH260233.1
India	Unknow	MH260226.1
Thailand	Unknow	MH260225.1
Pakistan	Unknow	MH260224.1
USA	Unknow	MH260221.1
Egypt	Unknow	MH260217.1
USA	Unknow	MH260210.1
Turkey	Unknow	MH260209.1
Vietnam: Hanoi	*Justicia gendarussa* (Acanthaceae)	KJ187588.1
USA: Carolina	Unknow	KJ187586.1
China: Hainan	*Hibiscus rosa-sinensis* (Malvaceae)	KJ187585.1
Pakistan: Baluchistan Naseer Abad district	*Gossipium hirsutum* (Malvaceae)	KJ995914.1
Philippines: Tarlac City	*Hibiscus rosa-sinensis* (Malvaceae)	KF442959.1
Vietnam: Ha Noi City	*Justicia gendarussa* (Acanthaceae)	KF442956.1
Pakistan: Multan City	*Hibiscus rosa-sinensis* (Malvaceae)	KF442955.1
China: Guangxi, Hubei, Guangdong	*Hibiscus rosa-sinensis* and *Gossipium hirsutum* (Malvaceae)	KJ620516.1
Israel: Arava Valley	*Hibiscus mutabilis* (Malvaceae)	KT369526.1
Turkey: Adana	*Lycopersicon esculentum* (Solanaceae)	KT369524.1
Pakistan	*Gossipium hirsutum* (Malvaceae)	AB499696.1
USA: Florida	Unknow	EU267208.1
